# Dimensionally Consistent Formation Pressure Prediction from Borehole Sensing Data Using Pressure Ratio Symbolic Regression

**DOI:** 10.3390/s26175440

**Published:** 2026-08-28

**Authors:** Chi Zhao, Ming Zhang, Lihao Zhou, Jin Wang, Wenxuan Kou, Guojie Liu

**Affiliations:** 1 College of Petroleum Engineering, Xi’an Shiyou University, Xi’an 710065, China; 2 Key Laboratory of Exploration and Development of Complex and Difficult-to-Produce Oil & Gas Reservoirs (Xi’an Petroleum University), Ministry of Education, Xi’an 710065, China; 3 Shaanxi Key Laboratory of Advanced Stimulation Technology for Oil & Gas Reservoirs, Xi’an 710065, China

**Keywords:** symbolic regression, formation pressure, equivalent mud weight, pressure ratio, dimensional consistency, external-well validation, local recalibration

## Abstract

Formation pressure labels are often stored as equivalent mud weight (EMW), whereas engineering analysis requires true pressure and validation strategies that account for the strong depth dependence of borehole data. This study develops a dimensionally consistent pressure ratio symbolic regression framework in which vertical overburden stress provides the pressure scale and a compact dimensionless correction is learned from reference-scaled borehole variables. After converting EMW-based labels to MPa and applying transparent quality control criteria, 1877 of 2025 depth-indexed records were retained. Redundant and deterministically derived variables were identified through correlation analysis, variance inflation factors, principal component analysis, and deterministic relation checks and were excluded from the compact symbolic search. Five contiguous depth blocks with a 20 m purge zone were used for leakage-resistant validation, with symbolic-structure selection repeated within each outer training fold. The selected four-coefficient expression, comprising an intercept and three predictor-dependent terms, retained only depth and acoustic slowness. Fixed-structure depth-block validation yielded a coefficient of determination (R^2^) of 0.3008, a root mean square error (RMSE) of 3.5439 MPa, a mean absolute error (MAE) of 2.6964 MPa, and a mean absolute relative error (MARE) of 10.02%, while the conservative nested symbolic pipeline yielded MARE = 11.60%. External zero-shot evaluation on 974 samples from an external well, 98.36% of which were outside the calibration depth range, yielded R^2^ = 0.0265, RMSE = 7.0024 MPa, MAE = 5.5723 MPa, and MARE = 11.51% against an independent D-exponent-based engineering pressure reference (PP_DX). Sparse local recalibration using 10 depth-spaced PP_DX engineering reference points, with ±20 m neighborhoods excluded from evaluation, improved performance to R^2^ = 0.8684, RMSE = 2.8431 MPa, MAE = 2.1108 MPa, and MARE = 4.39% on the remaining 564 samples. These results indicate that the proposed framework provides an explicit and independently auditable pressure equation that preserves the overall pressure scale under substantial depth domain shift and can be efficiently recalibrated using sparse local pressure information. Site-specific calibration remains necessary for accurate reproduction of local pressure variations.

## 1. Introduction

Formation pore pressure (hereafter formation pressure, Pp) is a fundamental input to well design, drilling fluid density selection, casing design, and well control planning. Inaccurate pressure estimates can narrow the safe operating window and increase the risks of influx, blowout, lost circulation, and wellbore instability, which can eventually increase drilling operation non-productive time and overall drilling cost [1,2,3,4,5,6].

Conventional formation pressure methods explain pressure variation through compaction and effective stress relationships. Eaton-type approaches use normal compaction trends from sonic, resistivity, or related indicators, whereas Bowers-type formulations relate velocity trends to effective stress and can account for overpressure mechanisms beyond simple undercompaction [1,2,3]. These methods are physically interpretable and remain valuable when normal-compaction trends and reliable pressure control points are available; however, unloading, diagenesis, lithological heterogeneity, and noisy measurements can weaken their calibration.

The operational literature also shows why pressure prediction should be connected to drilling data and well stability. Krygier et al. [5] examined non-productive time from a drilling company perspective and identified well stability problems as a practical source of time loss and cost. Elmgerbi and Thonhauser [6] assessed advanced sensors and the Internet of Things for anticipating downhole drilling issues, highlighting the value of timely multi-source sensing. These studies motivate a pressure workflow that is not only accurate on a calibration table but also auditable and usable when data conditions change.

Data-driven models address some of the nonlinearities that are difficult to encode with a single empirical transform. Random Forest aggregates decorrelated trees to model nonlinear interactions, while gradient-boosting and support vector methods provide flexible alternatives for tabular regression [7,8,9,10]. In pore pressure applications, Yu et al. [11] demonstrated that multivariate petrophysical information can capture nonlinear pressure responses, Abdelaal et al. [12] showed how drilling parameters can support pressure gradient prediction while drilling, and Zhang et al. [13] emphasized that feature quality and selection materially affect the reliability of log-based prediction.

Recent studies have extended this data-driven direction toward integrated, knowledge-guided, and real-time workflows. Li et al. [14] developed a comprehensive machine-learning approach for abnormally high-pressure blocks; Cao et al. [15] combined geological knowledge with a temporal fusion transformer; and Zhang et al. [16] introduced physics-informed deep learning using seismic data. Ehsan et al. [17] integrated conventional well logs and seismic attributes, whereas Chen et al. [18] embedded petrophysical theory into seismic and logging-while-drilling data for ahead-of-bit prediction. Other recent work has explored stacking with mud-log data [19], integrated well-logging and mud-logging inputs [20], and feature selection combined with physics-constrained attention networks [21]. Together, these studies improve nonlinear representation and multi-source integration, but their performance and transferability remain closely tied to field-specific data distributions, geological similarity, acquisition provenance, and validation design.

Symbolic regression offers a complementary objective: an explicit mathematical expression that can be inspected, independently implemented, and recalibrated. Schmidt and Lipson [22] demonstrated the potential of symbolic regression to recover compact relationships from data, while the AI Feynman framework of Udrescu and Tegmark [23] showed how physics-inspired decomposition can improve symbolic discovery. Physics-informed and theory-guided learning studies further demonstrated that physical knowledge can constrain data-driven models [24,25,26]. These findings support the use of explicit mathematical structure, but they do not by themselves ensure dimensional consistency, low redundancy, or reliable out-of-sample behavior.

Three methodological issues are therefore important. First, deterministic or highly collinear petrophysical variables can create misleading importance rankings and unnecessarily complex expressions; collinearity diagnostics and principal component analysis provide complementary ways to identify this redundancy [27,28]. Second, randomly mixing neighboring depth samples can overstate generalization because borehole records are strongly autocorrelated. Cross-validation studies for structured data show that the partition design must respect the dependence structure of the observations [29]. Third, interpretability means more than displaying a short formula: the preprocessing, feature construction, model selection process, and uncertainty of the result must also be auditable [30,31].

These concerns are especially important for depth-indexed borehole tables, where well provenance, acquisition information, and direct pressure control metadata may be incomplete.

Taken together, the literature leaves a specific methodological gap. Existing studies demonstrate the value of multivariate and physics-guided prediction, yet few workflows simultaneously control deterministic redundancy before symbolic search, evaluate the complete structure selection process under depth-aware partitions, and distinguish locked equation transfer from sparse local recalibration. This gap is especially relevant when well identifiers and direct pressure test metadata are unavailable, because random splits or unqualified cross-well claims can produce overly optimistic estimates of generalization [11,12,13,14,15,16,17,18,19,20,21,27,29].

This study addresses the gap by separating the dimensional pressure scale from a learned dimensionless correction. Vertical overburden stress supplies the pressure magnitude, while a compact symbolic function of reference-scaled borehole variables predicts the pressure ratio. The specific claim is deliberately bounded: the resulting equation is intended to be an auditable, dataset-calibrated pressure profile and screening model whose pressure scale can be tested under transfer and whose coefficients can be locally recalibrated, rather than a universal physical law.

The proposed workflow therefore combines (i) EMW-to-MPa conversion and transparent label screening; (ii) Pearson and Spearman correlation, deterministic relation checks, variance inflation factors (VIF), and principal component analysis (PCA); (iii) a compact symbolic library built from reference-scaled variables; (iv) leakage-resistant validation using contiguous depth blocks and nested model selection; and (v) a separate external-well transfer test followed by sparse local coefficient recalibration.

Accordingly, the study asks whether a short dimensionally consistent equation can achieve an error magnitude comparable to nested ensemble models, remain transparent enough for independent recalculation, and retain useful pressure-scale behavior when transferred to an external well with only sparse local recalibration.

The study proceeds through five linked stages:

(1) Audit the data, convert equivalent-density labels to pressure, disclose the active screening rules, and quantify feature redundancy;
(2) Formulate the dimensionless target λp = Pp/σv and construct reference-scaled candidate variables;
(3) Select one- to three-term expressions using a score that combines mean held-out MARE, worst-block MARE, and expression complexity;
(4) Compare the selected equation with repeated random splits, nested depth-block validation, controlled target ablations, nested black box models, and engineering baselines; and
(5) Evaluate the locked equation on the external well and quantify sparse local recalibration using depth-spaced PP_DX engineering-reference points with ±20 m purge neighborhoods.

The internal depth-block and external-well experiments are interpreted separately: the former quantify within-dataset generalization under depth-aware partitioning, whereas the latter quantify cross-dataset engineering reference transfer and the amount of local information needed for recalibration.

The main contributions are:

(1) A hybrid formulation Pp = σv f(π), in which σv provides the pressure scale and f(π) is dimensionless;
(2) A redundancy audit that prevents exact derived variables and severe multicollinearity from dominating symbolic search;
(3) Contiguous depth-block validation with a purge zone and nested structure selection to reduce adjacent-sample leakage and quantify model-selection uncertainty; and
(4) An external-well transfer experiment that reports the locked equation zero-shot result separately from 5-, 10-, and 20-point sparse local coefficient recalibration, with ±20 m neighborhoods removed from recalibration evaluation.

## 2. Pressure Ratio Symbolic Regression with Dimensional and Stability Constraints

### 2.1. Symbolic Regression and Expression Tree Representation

Expression trees provide a standard representation for symbolic regression and genetic programming [32,33]. Internal nodes contain mathematical operators, whereas terminal nodes contain input variables and numerical constants. This representation provides the conceptual basis for exploring mathematical expressions without prescribing a complete regression equation in advance.

Let *X* denote the model inputs, *F* the admissible function set, and *C* the numerical constants. A candidate symbolic model can be written as Equation (1):

(1) y=f(X,F,C)

where *y* is the predicted target and *f*(*X*,*F*,*C*) is the expression encoded by the GP tree.

**Figure 1:**
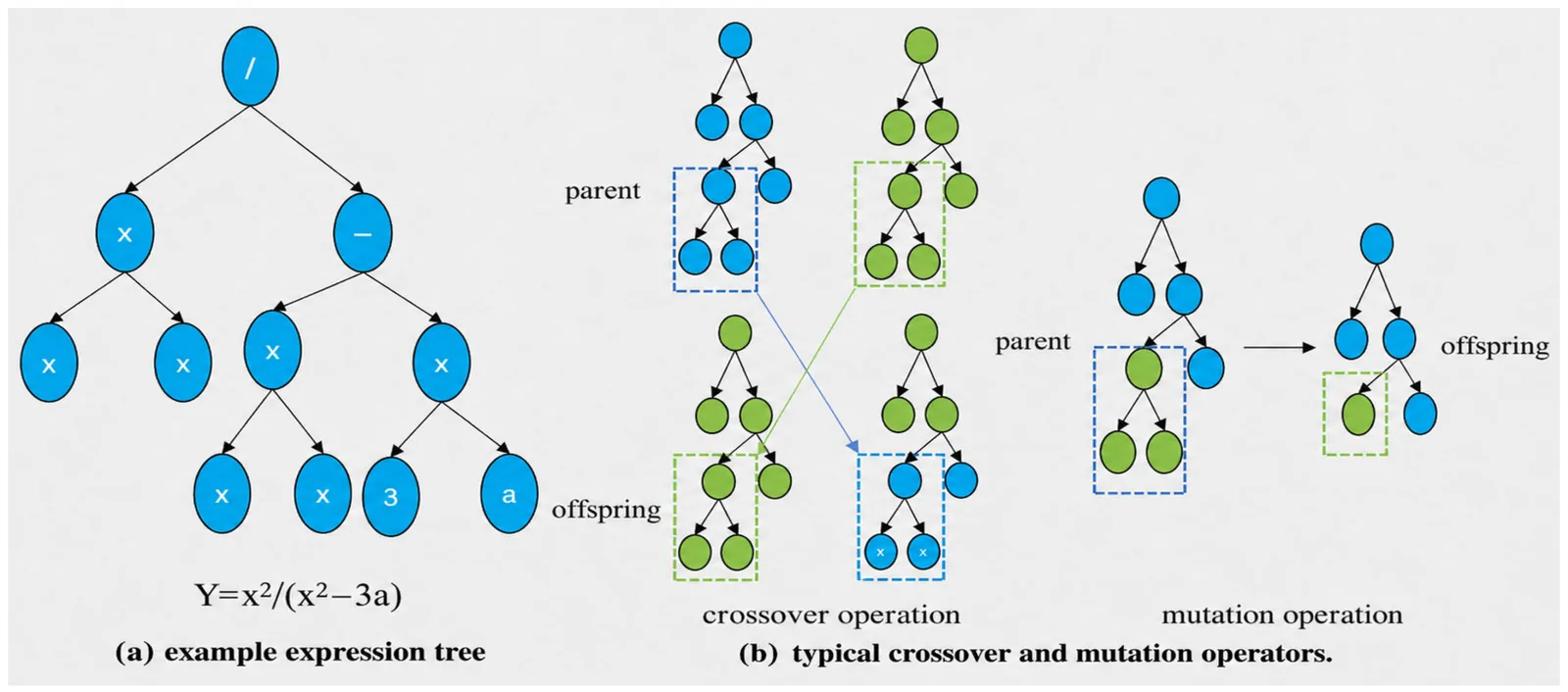
Conceptual symbolic regression representation. The symbol × denotes the multiplication operator. Blue and green nodes distinguish subtrees originating from different parent or replacement structures, while dashed boxes indicate the subtrees selected for crossover or mutation.

Figure 1 illustrates the general expression tree representation and typical crossover and mutation operators as conceptual background. The revised implementation used the compact candidate-term search described in Section 2.2.3 rather than an unrestricted genetic programming population.

Compared with a fitted ensemble, a symbolic expression can be evaluated without a serialized model object and can be inspected term by term [22,23]. This transparency is useful only when the search space, preprocessing, and validation protocol are also disclosed; a short equation is not, by itself, evidence of physical validity or transferability.

### 2.2. Pressure Ratio and Geological Constraint Design

Formation pressure evolves with burial, compaction, overburden loading, fluid expansion, diagenesis, and hydraulic communication. Instead of imposing unverified geological rules on every candidate expression, the revised framework uses three transparent forms of guidance: an explicit pressure scale, a dimensionless prediction target, and stability-based selection across held-out depth intervals.

Optional engineering output checks are evaluated after model selection and are not included in the primary fitness score or in the reported primary metrics.

#### 2.2.1. Pressure Scale and Pressure Ratio Formulation

The source columns Pp and σv are stored as equivalent-density values in g/cm^3^. They are converted to true pressure before modeling. The dimensionless target is λp = Pp/σv (Equation (3)), so σv provides the physical pressure magnitude and symbolic regression learns only the correction ratio.

#### 2.2.2. Unit Conversion and Engineering-Bound Treatment

For depth D in meters, an equivalent-density label ρeq in g/cm^3^ is converted to pressure using Equation (2):

P(MPa) = ρ_eq(g/cm^3^) × 0.00980665 × D(m) (2)

The conversion is applied independently to *Pp* and σv. The target and reconstruction are given by Equations (3) and (4):

(3) $$\lambda_{p}\;=\;\frac{P_{p}}{\sigma_{v}}$$

(4) $$P_{p}\left(MPa\right)=\sigma_{v}\left(MPa\right)\lambda_{p}$$

The former model embedded a 0.505 lower threshold and clipped *λ_p_* to [0.20, 1.05]. The revised symbolic equation contains neither value. The former interval is reported only as an output diagnostic and optional engineering sensitivity; it is not used in structure selection and is not applied to the primary validation metrics.

#### 2.2.3. Stability-Constrained Symbolic Search

Candidate terms were generated from reference-scaled depth, gamma ray, acoustic slowness, density, Poisson ratio, shale fraction, stress-normalized mechanical properties, and sin(φ). Exact deterministic derivatives and severe redundancies were removed before the final search. Each one- to three-term structure was refitted in the training portion of every depth block. The selection objective is defined in Equation (5).

(5) $$J=MARE_{mean}+0.30\;MARE_{worst}+0.06\;N_{op}+0.04\;N_{term}$$

where *MAREmean* is the mean held-out mean absolute relative error, *MAREworst* is the largest block-level *MARE*, and *Nop* and *Nterm* denote the number of operators and symbolic terms, respectively. The coefficients 0.30, 0.06, and 0.04 control the relative penalties for worst-block error and complexity. The candidate-term construction, scoring function, and selection procedure are described explicitly to support reproducibility.

This score discourages formulas that obtain low average error at the expense of one poorly predicted interval or excessive algebraic complexity. To quantify selection uncertainty, the complete structure selection procedure was also repeated inside each outer training fold.

**Figure 2:**
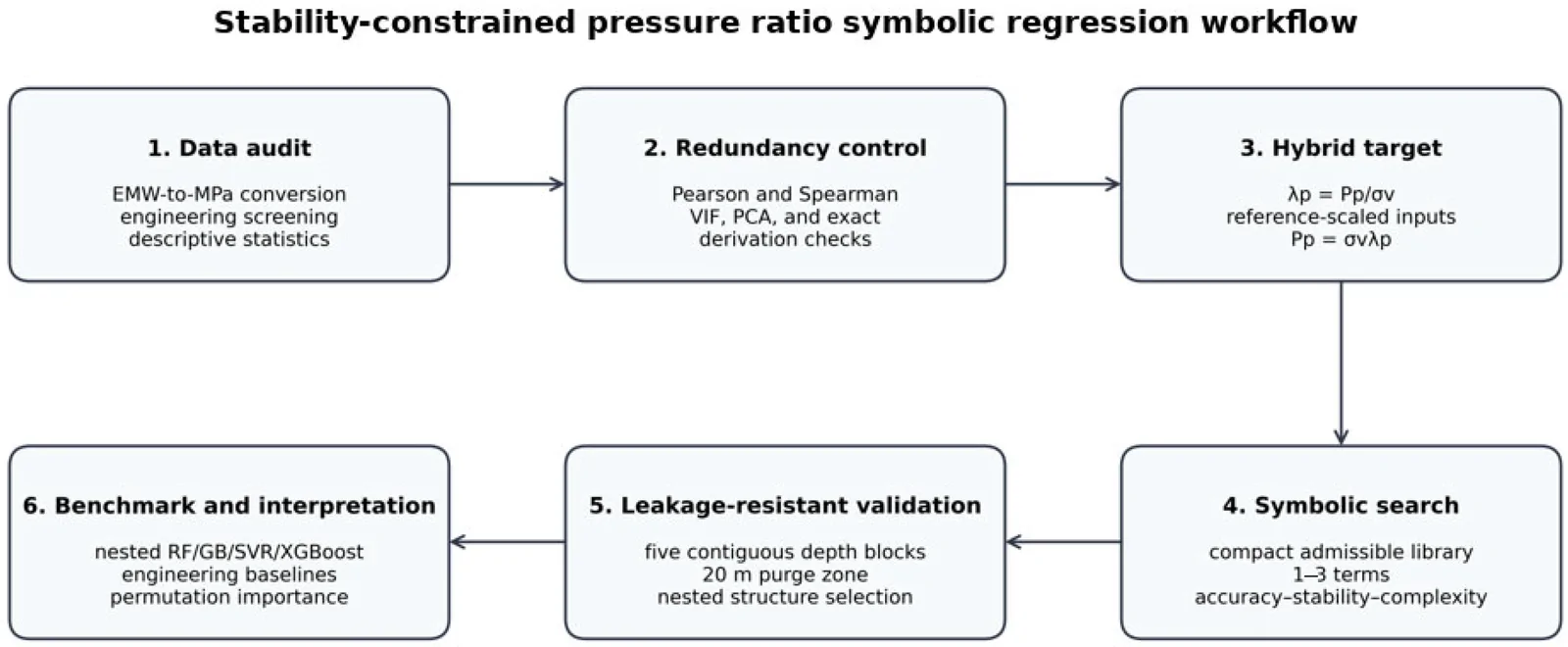
Workflow of the stability–constrained pressure ratio symbolic regression framework.

The workflow in Figure 2 makes the pressure scale, data audit, admissible search space, nested validation, and benchmark interpretation explicit.

### 2.3. Dimensional Consistency Constraint Design

Dimensional consistency is enforced by separating the pressure scale from the learned correction. The final model is given in Equation (6), where *λp* and every argument supplied to the symbolic function are dimensionless.

Reference-scaled candidates included $\pi_{D}\;=\;\frac{D}{1000\;m}$, $\pi GR\;=\;\frac{GR}{100API}$, $\pi DT\;=\;\frac{DTc}{\frac{100\;\mu s}{f_{t}}}$, $\pi_{\rho }\;=\frac{\;\rho }{2.65\;g/cm^{3}}$, the dimensionless Poisson ratio and shale fraction, stress ratios such as $\frac{E}{\sigma v}$, $\frac{UCS}{\sigma v}$, and $\frac{C}{\sigma v}$, and sinφ. The reference values are fixed unit scales, not fitted thresholds.

Deterministic checks showed that $V_{p}=\frac{304.8}{DTc}$, *Vs* is determined by *Vp* and Poisson ratio, and *E* is determined by density, *Vs*, and Poisson ratio to numerical precision. *Vsh* is derived primarily from *GR*, whereas *UCS* and *C* are highly correlated. These variables were retained for descriptive auditing but were not allowed to enter the compact symbolic grammar as independent evidence.

(6) $$P_{p}\left(MPa\right)=\sigma_{v}\left(MPa\right)*f\left(\pi D,\;\pi GR,\;\pi DT,\;\pi \rho ,\;\pi PR,\;\pi Vsh,\;\frac{\pi E}{\sigma_{v}},\;\frac{\pi UCS}{\sigma_{v}},\;\frac{\pi C}{\sigma_{v}},\;sin\varphi \right)$$

The symbolic function $f\left(\pi D,\;\pi GR,\;\pi DT,\;\pi \rho ,\;\pi PR,\;\pi Vsh,\;\frac{\pi E}{\sigma_{v}},\;\frac{\pi UCS}{\sigma_{v}},\;\frac{\pi C}{\sigma_{v}},\;sin\varphi \right)$ returns *λp* and is dimensionless.

Pearson and Spearman correlation, *VIF*, *PCA*, and deterministic-relation checks were used as diagnostics rather than as final symbolic coordinates [27,28]. The first two principal components explained 93.83% of total variance, and the first five explained 99.55%, confirming extensive redundancy.

The selected equation ultimately retained only *πD* and *πDT*. Its numerical coefficients are calibrated to the primary dataset and should be locally re-estimated when reliable reference data from a different field or data source are available; the final printed form is given in Equation (11).

Thus, dimensional consistency is imposed by construction, whereas predictive stability and simplicity are controlled by held-out depth-block error and explicit complexity penalties.

## 3. Model Construction and Experimental Design

### 3.1. Data Sources and Preprocessing

#### 3.1.1. Data Sources

The primary calibration file contains 2025 depth-indexed records and 21 columns. The raw depth interval is 1188–3215 m. This primary table does not contain well IDs, block IDs, formation names, lithology counts, acquisition tool fields, or calibration standard fields. It is therefore described as an anonymized post-processed borehole dataset containing measured and derived rock-property variables; no claim is made that all records were acquired by logging while drilling or that the internal depth blocks represent independent wells or blocks.

After conversion and engineering screening, 1877 records were retained from the primary dataset. Because adjacent depth records are strongly autocorrelated, the primary internal validation used five contiguous depth blocks with a 20 m purge zone on both sides of each held-out interval. The original random 80:20 split was retained only as a secondary diagnostic analysis over 100 random seeds.

#### 3.1.2. Data Preprocessing and Feature Auditing

Preprocessing comprised four operations: (1) independent conversion of Pp and σv from equivalent-density labels to MPa; (2) pre-specified quality control guards of 0.80–2.50 g/cm^3^ for Pp, 1.50–3.00 g/cm^3^ for σv, and 0.20–1.05 for λp; (3) finite-value checks; and (4) fitting every scaler, feature selection step, and model using only the corresponding training records. After screening, 1877 records were retained.

Feature auditing combined Pearson and Spearman correlation, VIF, PCA, and deterministic-equation checks. Vp and Vs had effectively infinite VIF because of exact calculation relationships; UCS, PR, depth, σv and E also showed very large VIF values. PCA showed that the first two components explained 93.83% of the variance and the first five explained 99.55%. These results support the use of reduced physical grammar rather than all 13 correlated variables [27,28].

All 148 excluded rows failed the lower Pp-EMW quality control limit of 0.80 g/cm^3^; 14 of those rows also had λp < 0.20. No record was removed by the upper Pp, σv, or λp limits. The upper guards therefore did not truncate the retained label distribution. These screening values are dataset-specific quality control settings and are not transferred as symbolic-model parameters.

**Figure 3:**
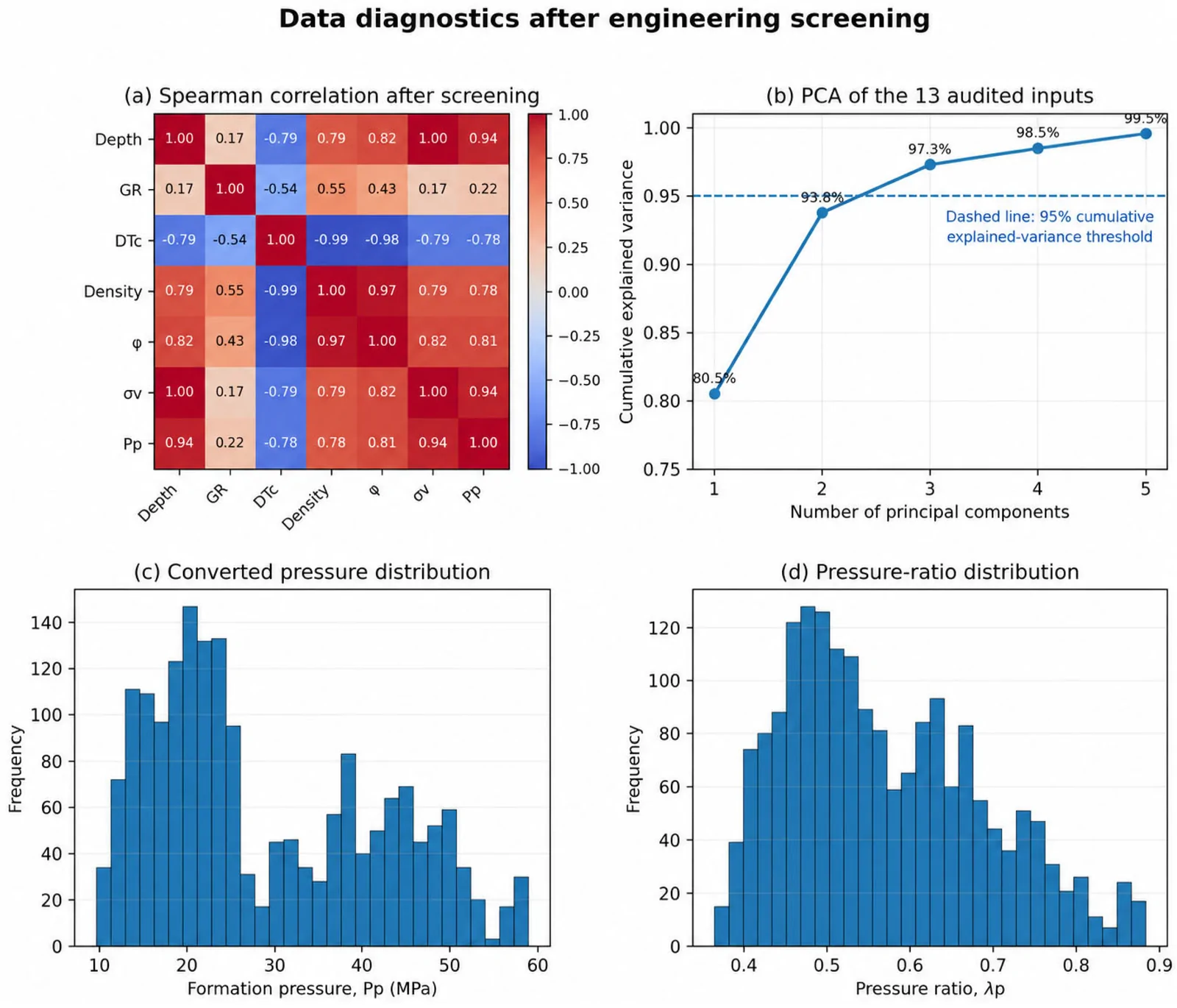
Data diagnostics after engineering screening: (**a**) Spearman correlation among the reduced physical variables; (**b**) PCA cumulative explained variance; (**c**) converted formation-pressure distribution; and (**d**) pressure ratio distribution. The dashed line in panel (**b**) denotes the 95% cumulative explained-variance threshold.

The retained samples had a mean depth of 2249.19 ± 572.63 m, mean Pp 28.87 ± 12.92 MPa, mean σv 48.84 ± 13.92 MPa, and mean λp 0.5701 ± 0.1184. Lag-1 correlations were approximately 0.987 for Pp, 0.931 for λp, and 0.953 for density, explaining why a shuffled split can overestimate generalization. Figure 3 summarizes the correlation, PCA, and distribution diagnostics; Table 1 reports descriptive statistics and the revised modeling role of every audited input.

The source-derived Vsh values were retained for descriptive auditing without post hoc clipping. Values outside the nominal interval [0, 1] reflect limitations of the original empirical derivation. Vsh was excluded from the compact symbolic grammar and therefore did not enter the final symbolic equation.

#### 3.1.3. External-Well Engineering Reference Dataset

A separate external-well table was used only for transfer evaluation after the symbolic structure and the original four coefficients had been fixed from the primary dataset. After retaining rows with finite depth, acoustic slowness (DT), overburden gradient (OBG), and PP_DX, 974 external samples remained over 3200–4173 m. No external-well sample was used for symbolic-structure discovery, original coefficient estimation, screening-limit selection, or internal hyperparameter tuning.

The external DT column is in μs/ft and was used as the acoustic-slowness input required by the selected equation. OBG is stored in MPa/100 m; it was converted to overburden-equivalent density as OBG/0.980665 and then to σv in MPa using the same depth-dependent pressure conversion applied to the primary data. PP_DX, a D-exponent-based engineering pore-pressure reference, was used as the external evaluation target. PP_DT was retained only as a diagnostic and was not used as the validation target because it is itself derived from acoustic slowness and would create circularity in the evaluation of a DT-based symbolic equation.

### 3.2. Construction of the Stability-Constrained Pressure Ratio Model

The revised model construction consists of five steps: (1) convert equivalent-density labels to MPa and calculate λp; (2) audit redundancy and construct reference-scaled dimensionless variables; (3) generate a compact symbolic library containing arithmetic, square-root, logarithmic, reciprocal, product and ratio terms; (4) evaluate one- to three-term structures using the accuracy–stability–complexity score across contiguous depth blocks; and (5) refit the selected structure on the available calibration data. The complete structure-selection procedure is repeated inside the outer training folds for nested validation.

### 3.3. Experimental Design and Evaluation Metrics

#### 3.3.1. Experimental Protocol

Six complementary experiment groups were used to evaluate accuracy, stability, interpretability, transfer behavior, and engineering relevance.

(1) Repeated random-split analysis: 100 random 80:20 splits were used only to quantify sensitivity to the random seed and to show the optimism associated with adjacent-sample mixing.
(2) Primary depth-block validation: five contiguous depth intervals were held out in turn, with a 20 m purge zone excluding neighboring records from the training set [29].
(3) Nested symbolic validation: candidate structures were selected using only each outer training set and then evaluated on the untouched outer depth block.
(4) Target and model comparison: EMW, direct Pp in MPa, and λp were compared; RF, Gradient Boosting, XGBoost, and SVR were tuned only within each outer training fold and evaluated on the same held-out intervals.
(5) Engineering and ablation baselines: the former clipped-density expression, hydrostatic pressure, and an Eaton-type reference were evaluated under the same depth blocks.
(6) External-well transfer and sparse local recalibration: the locked equation was evaluated zero-shot on the external well, after which only the four numerical coefficients of the fixed symbolic structure were re-estimated from 5, 10, or 20 depth-spaced PP_DX reference points. The model selection procedure, random-state controls, and computational environment are described in the manuscript to support reproducibility.

#### 3.3.2. Evaluation Metrics

Let *y_i_* denote the measured formation pressure in MPa, *ŷ_i_* the predicted formation pressure in MPa, *ȳ* the mean measured formation pressure, and *n* the number of held-out records. Model performance was evaluated using the coefficient of determination (*R^2^*), root mean square error (*RMSE*), mean absolute error (*MAE*), and mean absolute relative error (*MARE*), defined in Equations (7)–(10):

(7) $$R^{2}\;=\;1\;-\;\frac{\sum_{i=1}^{n}{\left(y_{i}-{\hat{y}}_{i}\right)}^{2}}{\sum_{i=1}^{n}{\left(y_{i}-\bar{y}\right)}^{2}}$$

(8) $$RMSE=\sqrt{\;\frac{1}{n}\sum_{i=1}^{n}{\left({\hat{y}}_{i}-y_{i}\right)}^{2}}$$

(9) $$MAE=\frac{1}{n}\sum_{i=1}^{n}\left|{\hat{y}}_{i}-y_{i}\right|$$

(10) $$MARE(\%)=\frac{100}{n}\sum_{i=1}^{n}\frac{\left|{\hat{y}}_{i}-y_{i}\right|}{\left|y_{i}\right|}$$

All retained formation pressure values were positive; therefore, *|y_i_|* provided an unambiguous denominator for *MARE*. Fold-level results, aggregate statistics, and the worst-fold *MARE* are reported where applicable.

#### 3.3.3. Baseline Models

Four nonlinear regressors were evaluated as accuracy references using the same λp target, outer depth blocks, purge distance, and training-fold-only hyperparameter selection.

(1) Random Forest (RF)

Random Forest aggregates decorrelated decision trees trained on bootstrap samples and random feature subsets [7]. It was included because it is robust to nonlinear interactions and tabular predictor distributions.

(2) Support Vector Regression (SVR)

Support vector regression maps inputs through a kernel-defined feature space and controls model complexity through the margin and regularization parameters [9]. It provides a contrasting non-tree nonlinear baseline for a dataset of this size.

(3) Gradient Boosting and Extreme Gradient Boosting (XGBoost)

Gradient Boosting and XGBoost model nonlinear interactions through sequential residual correction; XGBoost additionally incorporates explicit regularization and efficient tree construction [8,10].

All four black-box models predicted λp and were reconstructed as Pp = σvλp using Equation (4). Hyperparameters were selected exclusively through inner depth-block validation within each outer training set; the outer held-out block was never used for hyperparameter or model selection. The candidate search spaces, fixed random-state settings, and preprocessing rules are reported in Appendix A, Table A1. Consequently, all comparisons used the same target, purge distance, and leakage-resistant outer test intervals.

#### 3.3.4. External-Well Validation and Sparse Local Recalibration

Zero-shot external evaluation used the printed four-coefficient equation exactly as obtained from the 1877-record primary calibration dataset. No external row was used to alter the symbolic structure or the original coefficients, and no λp clipping was applied to the primary external metrics. R^2^, RMSE, MAE, MARE, Pearson correlation, and mean prediction bias were calculated against PP_DX.

For sparse local recalibration, target depths were evenly spaced over the external interval while excluding the two interval endpoints, and the nearest unused observed row was selected for each target. Sets of 5, 10, and 20 PP_DX engineering reference points were examined. At the selected points, a local reference pressure ratio was calculated as λp,ref = PP_DX/σv, and only the four numerical coefficients of the fixed symbolic structure were re-estimated by least squares; the symbolic structure itself was not changed. The recalibrated coefficients are therefore treated as numerical adaptation parameters rather than new physical constants.

To reduce adjacent-depth leakage, every row within ±20 m of any external calibration point was excluded from the corresponding recalibration evaluation. The 10-point setting was used as the primary reported recalibration because it retained a substantial independent evaluation set, whereas the 5- and 20-point settings were retained as sensitivity analyses. Zero-shot and recalibrated results are reported separately.

## 4. Experimental Results and Analysis

### 4.1. Optimal Expression and Physical Interpretation

The stability-constrained search selected the following compact pressure ratio expression. Together with the pressure reconstruction in Equation (4), the fitted expression is reported in Equation (11).

Equation (11) comprises an intercept and three predictor-dependent symbolic terms (four fitted coefficients) and can be evaluated directly in a spreadsheet or short script without a stored model object.

The structure combines a burial-depth trend with an acoustic-slowness correction. Overburden stress is not counted as a learned importance term because it already supplies the pressure scale through Equation (4). GR, density, and rock-strength variables can still contribute to flexible black-box models, but they were not retained consistently once symbolic stability and complexity were penalized.

The expression is therefore interpreted as a dataset-calibrated depth–acoustic correction, not as a universal physical law. Its explicit form permits transparent recalibration when independent local pressure reference data become available.

(11) $$\lambda_{p}=-1.24804139-0.32596862\frac{D}{1000}+1.65339204\sqrt{\frac{DTc}{100}}+0.36778248\left(\frac{\frac{D}{1000}}{\frac{DTc}{100}}\right)$$

Here, D is in meters, and *DTc* is in μs/ft. The final ratio term is outside the square root. Independent refitting of the fixed four-coefficient structure on all 1877 retained records changed no printed coefficient by more than 1.4 × 10^−8^. The corrected printed equation yields a full-data fitted λp range of 0.4475–1.0804.

### 4.2. Accuracy and Robustness Verification Results

With the symbolic structure fixed and refitted within each of the five training partitions, the model achieved mean R^2^ = 0.3008 ± 0.1798, RMSE = 3.5439 ± 1.3348 MPa, MAE = 2.6964 MPa, and MARE = 10.02 ± 1.40%. When the complete symbolic-structure selection was repeated within each outer training fold, the conservative nested estimate was R^2^ = −0.1492, RMSE = 4.6678 MPa, MAE = 3.3997 MPa, and MARE = 11.60%. Table 2 summarizes the fixed-structure, nested-selection, and shuffled-split estimates.

The 100 shuffled 80:20 splits produced R^2^ = 0.9249 ± 0.0089 and MARE = 9.45 ± 0.37%; RMSE = 3.5235 MPa and MAE = 2.5427 MPa are reported as arithmetic means. However, 94.4% of the original random-test samples were only 1 m from a training sample. These values are therefore reported as a leakage diagnostic rather than as the primary generalization estimate.

The much higher shuffled-split R^2^ reflects the preservation of the global depth trend when adjacent samples are mixed across training and test sets. By contrast, block-wise R^2^ evaluates the ability to reproduce variation within a withheld interval and is intentionally more demanding.

**Figure 4:**
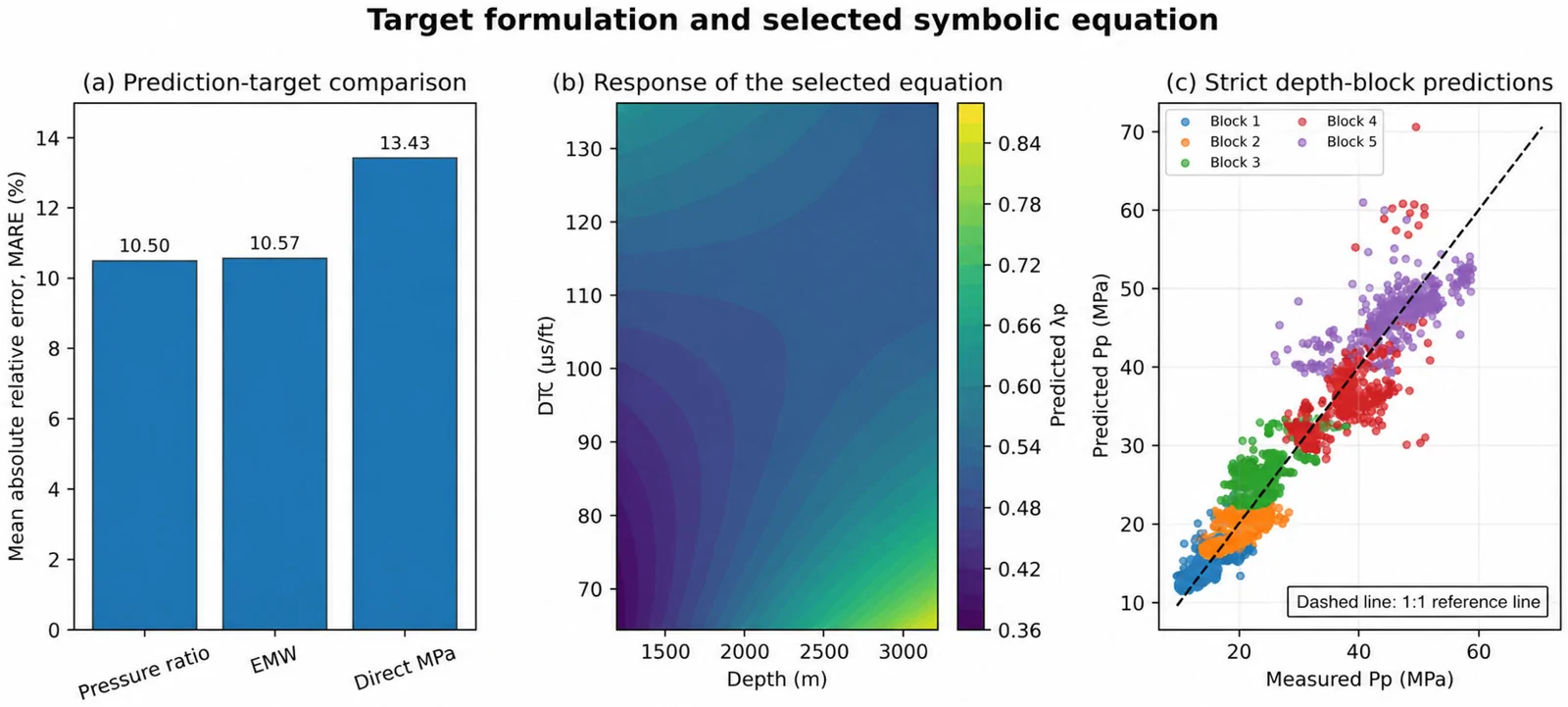
Target formulation and selected equation: (**a**) controlled target comparison; (**b**) response surface of the selected equation; and (**c**) strict depth-block predictions. The dashed line in panel (**c**) denotes the 1:1 reference line.

Figure 4 compares the three target formulations, visualizes the response of the selected equation, and shows measured versus predicted Pp for the strict depth-block analysis.

**Figure 5:**
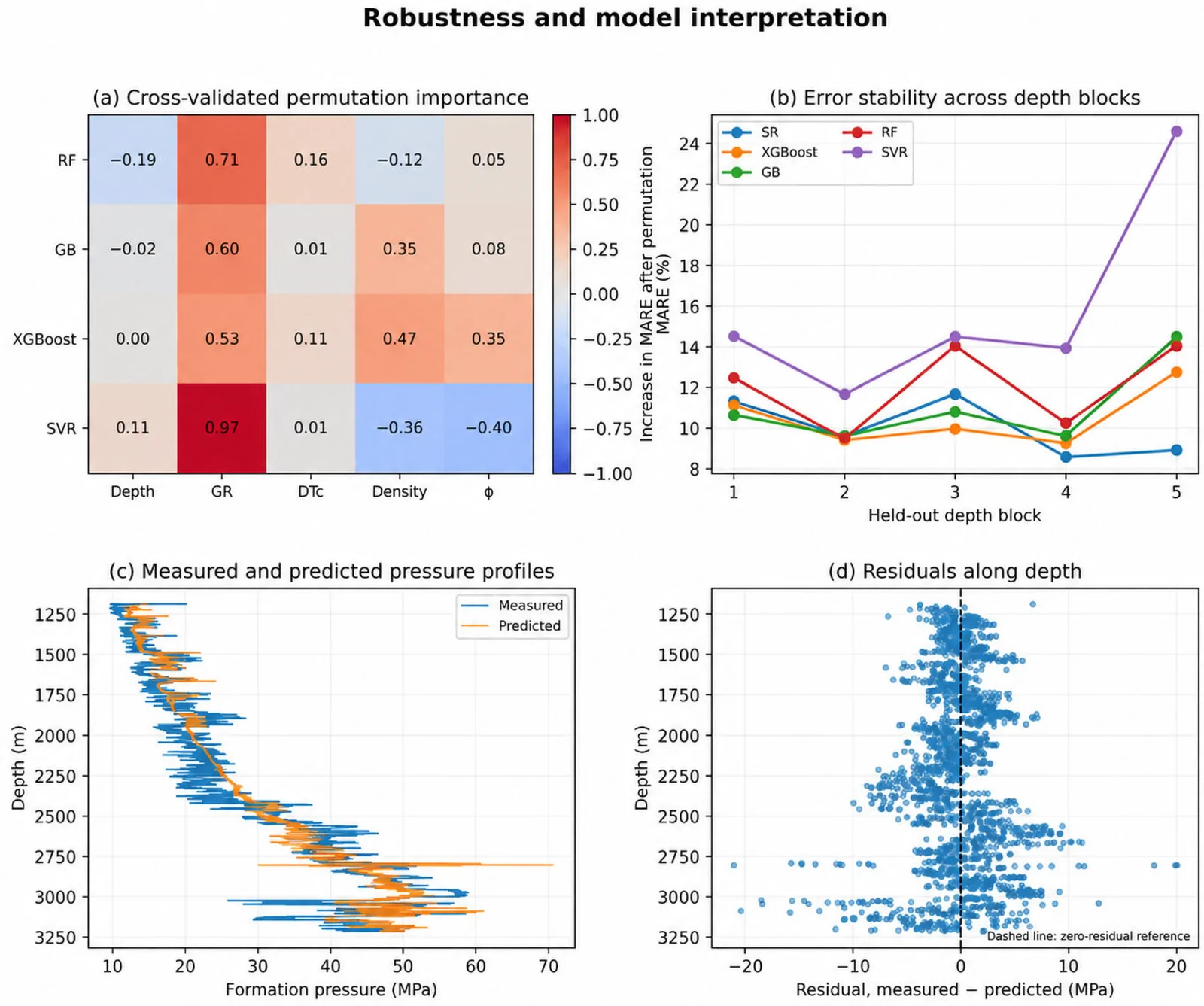
Robustness and interpretation: (**a**) cross−validated permutation importance; (**b**) fold−wise MARE; (**c**) measured and predicted pressure profiles; and (**d**) residuals along depth. The dashed line in panel (**d**) denotes the zero-residual reference line.

Residuals remain depth-structured in several held-out intervals, particularly at greater depth. Figure 5 therefore presents both fold-wise MARE and the pressure/residual profiles rather than relying on a smoothed curve. Because RMSE exceeds MAE under both the fixed-structure and nested evaluations, a subset of held-out samples contributes comparatively larger errors; the model therefore does not fully capture local nonlinear variation.

### 4.3. Leakage-Resistant Depth-Block Validation Results

The retained interval was divided into five contiguous blocks: 1188–1671 m, 1672–2064 m, 2065–2464 m, 2465–2839 m, and 2840–3215 m. Records within 20 m of each held-out block were excluded from the corresponding training set.

For the fixed selected structure, fold MARE values were 11.33%, 9.61%, 11.67%, 8.58%, and 8.92%; the corresponding R^2^ values ranged from 0.0638 to 0.4739. For the complete nested symbolic-selection procedure, the worst outer-fold MARE was 12.72%. The complete fold-wise values are reported in Table 3.

R^2^ was calculated separately within each depth block and is therefore sensitive to the local variance of that block. The modest or negative nested R^2^ values show that local within-block variation is not fully represented, even when the scale-normalized absolute error remains approximately 10–12%. Both statistics are retained to avoid an overgeneralized claim of cross-block stability.

Because the source table lacks well and block identifiers, these experiments are described explicitly as within-dataset depth-block validation. They are not leave-one-well-out or cross-block validation.

### 4.4. Constraint and Target Validity Verification

Under the same five depth blocks, the former clipped-density expression achieved mean R^2^ = −0.8264, RMSE = 5.2921 MPa, and MARE = 16.83%. The selected pressure ratio equation reduced MARE by 6.81 percentage points. In the controlled target comparison, the best λp model achieved 10.50% MARE, the best EMW model 10.57%, and the best direct-MPa model 13.43%. Table 4 summarizes the constraint and target-ablation results.

These results support the pressure ratio formulation and show that the former 0.505 threshold was unnecessary for competitive error. The output interval [0.20, 1.05] was neither a symbolic term nor part of the final fitness score; it was retained only as a reported engineering sensitivity.

### 4.5. Multi-Model Comparison Results

**Figure 6:**
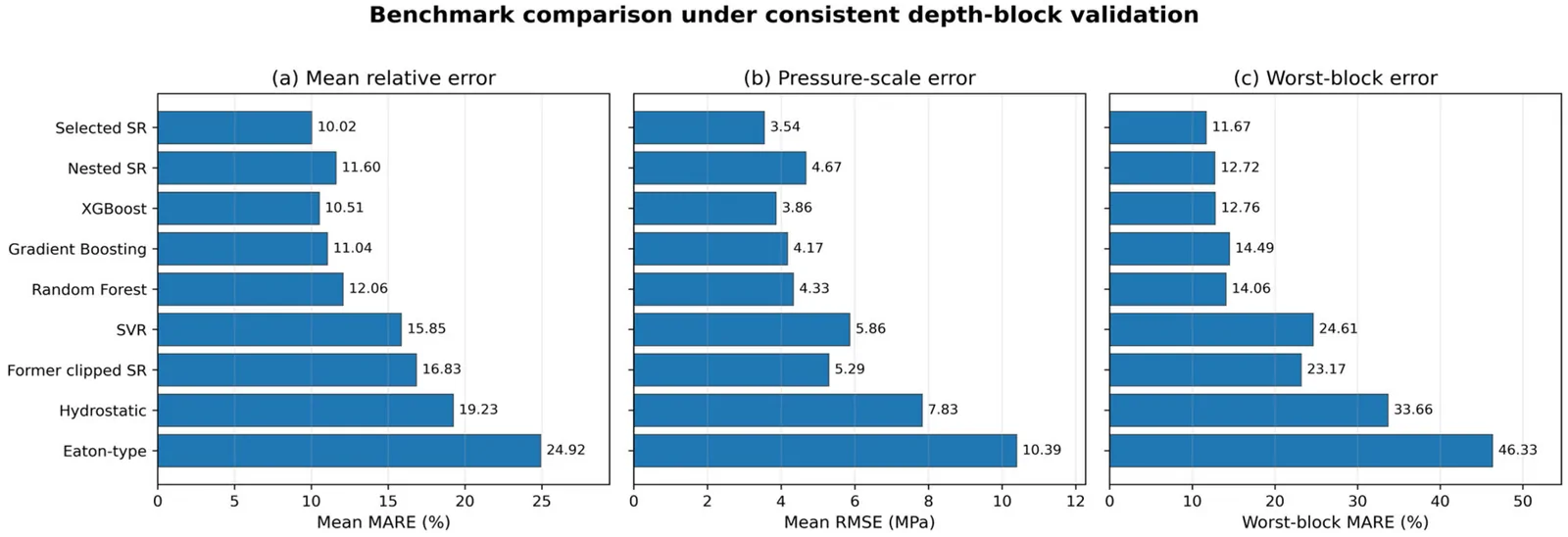
Benchmark comparison under consistent depth-block validation.

Table 5 and Figure 6 compare the revised symbolic model, nested black-box regressors, and engineering baselines under the same depth-block protocol.

Under fully nested tuning, XGBoost achieved the lowest black-box MARE (10.51%), followed by Gradient Boosting (11.04%), Random Forest (12.06%), and SVR (15.85%). The selected symbolic structure achieved 10.02% MARE when its form was fixed, whereas the complete nested symbolic-selection procedure achieved 11.60%.

The study therefore does not claim that symbolic regression is uniformly more accurate than black-box learning. Its principal advantage is an explicit auditable equation whose error is of the same order as the strongest nested ensembles.

The symbolic equation can be recalculated without a serialized estimator, whereas RF, Gradient Boosting, XGBoost, and SVR require the fitted model and preprocessing objects. Fold-wise permutation analysis of the compared black-box models indicated that GR and density could improve prediction, although their contributions varied across models and held-out intervals. Negative permutation values were treated cautiously because correlated predictors can make such estimates unstable.

For the selected symbolic structure, the pressure error corresponds to a mean absolute EMW error of approximately 0.120 g/cm^3^. About 52.1% of held-out records were within ±0.10 g/cm^3^ and 82.2% within ±0.20 g/cm^3^; 50.0% were within ±2 MPa and 79.2% within ±4 MPa. The 90th- and 95th-percentile absolute pressure errors were 6.08 and 7.56 MPa, respectively. These values support regional screening and locally calibrated profile estimation, but not the use of the equation alone for a narrow mud-weight window or well-control decision.

### 4.6. Sensitivity, Computational Cost, and Engineering Error

For the fixed selected structure, four to six depth blocks and purge distances of 0–30 m produced mean MARE values of approximately 9.64–10.04%, indicating limited sensitivity to those validation settings. Timings were measured in the revision environment on Linux using five AMD EPYC 9V74 CPU cores, Python 3.13.5, scikit-learn 1.8.0, and XGBoost 3.1.3, without GPU acceleration. Total nested tuning required approximately 2.08 s for XGBoost, 2.02 s for SVR, 10.41 s for Random Forest, and 13.19 s for Gradient Boosting. The calibrated symbolic equation evaluates essentially instantaneously. These timings are comparative and will vary with hardware and software versions.

### 4.7. External-Well Transfer and Sparse Local Recalibration

The locked equation was applied without refitting to 974 external-well samples. The external interval spans 3200–4173 m, and 98.36% of the samples lie outside the 1188–3215 m calibration depth range. Against the independent PP_DX engineering reference, the zero-shot equation yielded R^2^ = 0.0265, RMSE = 7.0024 MPa, MAE = 5.5723 MPa, MARE = 11.51%, Pearson r = 0.5987, and a mean prediction bias of −0.3833 MPa. The low zero-shot R^2^ indicates limited reproduction of local within-well pressure variation, even though the overall relative-error scale remained close to the 11.60% MARE obtained by the conservative nested symbolic pipeline.

Sparse local coefficient recalibration substantially reduced the external error while leaving the symbolic structure unchanged. With 5 reference points, 769 non-neighboring samples remained for evaluation and MARE decreased to 5.65%. With 10 reference points, 564 evaluation samples remained, and performance improved to R^2^ = 0.8684, RMSE = 2.8431 MPa, MAE = 2.1108 MPa, and MARE = 4.39%. A 20-point recalibration further reduced MARE to 3.83%, but the ±20 m purge zones left only 154 evaluation samples. The 10-point setting therefore provides the clearest balance between sparse local information and retained evaluation coverage. Table 6 summarizes the complete sensitivity analysis.

**Figure 7:**
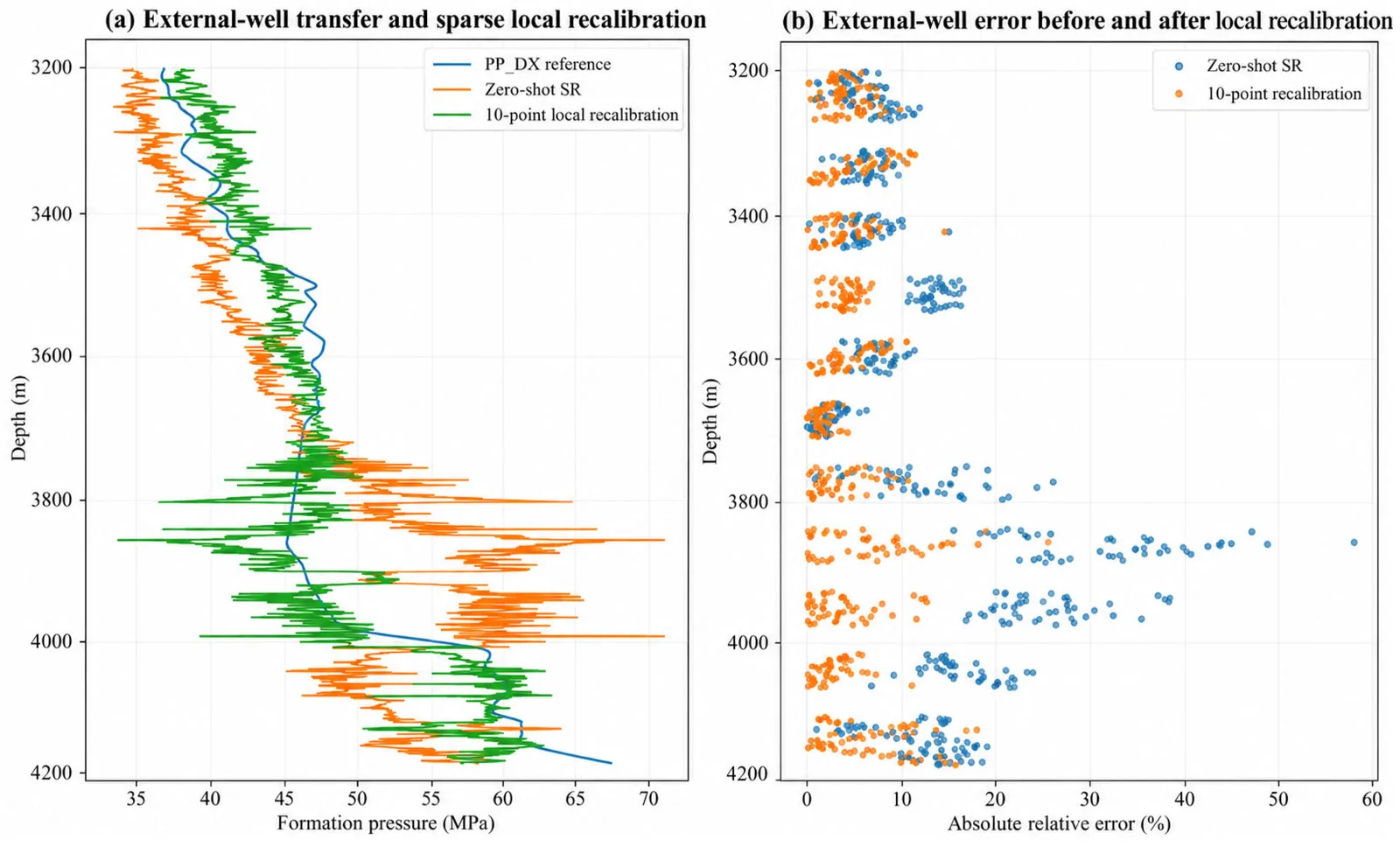
External-well transfer and 10-point sparse local recalibration: (**a**) PP_DX reference, zero-shot symbolic prediction, and locally recalibrated pressure profile; (**b**) absolute relative error before and after local recalibration.

Figure 7 compares the external pressure profile and depth-wise absolute relative error before and after the 10-point recalibration. The largest zero-shot deviations are concentrated mainly in the deeper part of the external interval, particularly around 3800–4000 m, whereas the locally recalibrated profile follows the PP_DX reference more closely over most of the well.

**Figure 8:**
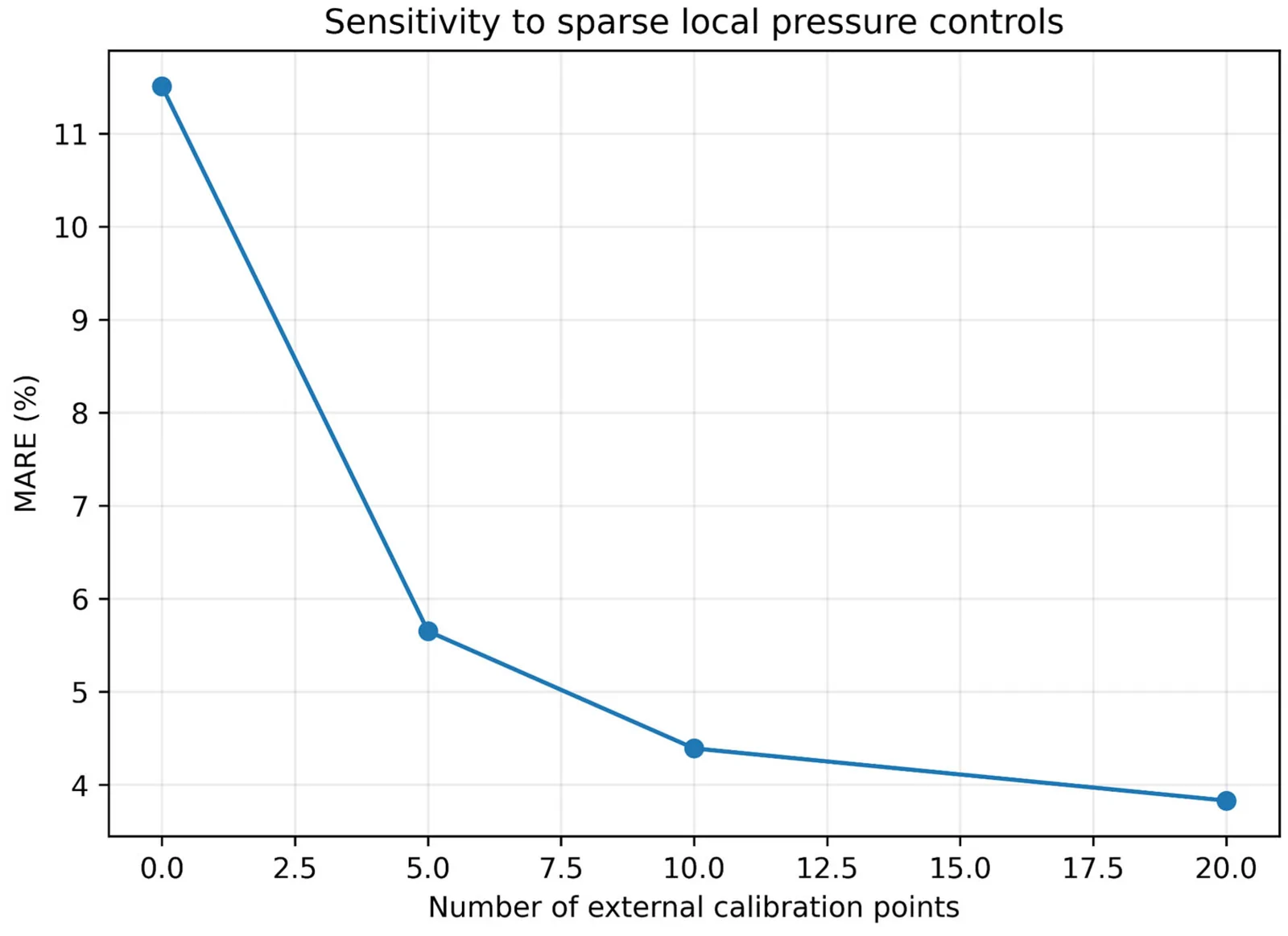
Sensitivity of external-well MARE to the number of depth-spaced local PP_DX engineering-reference points.

Figure 8 shows that most of the improvement occurs after introducing the first few local reference points, while additional calibration points provide progressively smaller reductions in MARE and simultaneously reduce the size of the purge-filtered evaluation set.

## 5. Discussion

### 5.1. Model Advantages and Engineering Significance

The revised experiments show that a pressure ratio symbolic model can provide a compact, auditable pressure equation while avoiding the optimistic conclusions of the original shuffled split. Relative error is substantially lower than that of the former clipped-density formula, although the conservative nested R^2^ remains modest in some held-out intervals. The external-well experiment further shows that the locked equation can retain the overall pressure scale under a substantial depth-domain shift, but that local pressure variation may require sparse site-specific adaptation.

The improvement is associated with five linked changes: explicit conversion of equivalent-density labels to MPa, separation of the pressure scale through the pressure ratio, removal of deterministic and severely collinear inputs, model selection based on contiguous held-out intervals rather than one shuffled partition, and explicit separation of external zero-shot transfer from local coefficient recalibration.

### 5.2. Model Performance and Interpretability

XGBoost achieved the lowest fully nested black-box MARE, and Gradient Boosting also remained competitive. Their predictions, however, require fitted estimators and preprocessing objects and cannot be reduced to a short field equation.

The symbolic result is therefore an interpretability–accuracy trade-off, not evidence that constrained symbolic regression always generalizes better than ensemble learning.

The selected equation can be inspected term by term, independently reimplemented, and recalibrated with sparse local pressure reference points. This transparency is valuable for auditing and sensitivity analysis and complements post-hoc explanation approaches for black-box models [22,30,31], but it does not remove the need for representative calibration data. In the external-well test, the zero-shot R^2^ of 0.0265 showed that the original coefficients did not reproduce all local pressure variation, whereas 10-point coefficient recalibration reduced MARE from 11.51% to 4.39% on 564 purge-filtered evaluation samples.

Where reliable normal-compaction trends and direct pressure-control points are available, locally calibrated Eaton- or Bowers-type methods may be preferable because their geological assumptions and calibration basis can be evaluated directly. The present symbolic equation should therefore be viewed as an auditable data-calibrated alternative rather than a universal replacement for those methods.

The external experiment also clarifies the meaning of “recalibration” in this study. Only the four numerical coefficients of the pre-existing symbolic structure are adjusted; the depth–acoustic structure itself is not rediscovered. Because these basis terms can remain correlated over a single well, the recalibrated coefficients are treated only as numerical local adaptation parameters and are not assigned separate physical interpretations.

### 5.3. Model Limitations

The present results should be interpreted as evidence for an auditable, dataset-calibrated workflow rather than a universal pressure law. Although the pressure-scale/data-correction decomposition provides a transferable modeling template, the selected terms, coefficients, and screening thresholds are specific to the calibration data and must be re-estimated and validated for each new region or acquisition setting.

The primary calibration table contains no well ID, block ID, lithology label, acquisition tool record, or direct pressure-test identifier. Accordingly, its five depth-block experiments quantify within-dataset depth-aware generalization, not leave-one-well-out or cross-block performance.

The external-well test partially addresses transferability but uses PP_DX, a D-exponent-based engineering pressure reference, rather than a direct MDT/RFT/DST measurement. The low zero-shot external R^2^ indicates that local pressure variation is not fully transferable without site-specific information.

Additional limitations are that the label-screening thresholds and numerical equation coefficients are dataset calibrated, and the uncertainty of the predicted profile has not yet been quantified probabilistically. The fixed symbolic structure was recalibrated only through its four numerical coefficients; it was not rediscovered on the external well, so the recalibrated coefficients are numerical adaptation parameters rather than new physical constants.

Until validation across multiple identified wells, direct pressure measurements, known acquisition provenance, and calibrated uncertainty intervals are available, the equation should be used as a locally recalibrated auxiliary screening and pressure profile tool rather than transferred unchanged for operational decisions.

## 6. Conclusions

This study reformulated the original symbolic regression workflow by converting equivalent-density labels to true pressure, predicting a dimensionless pressure ratio, removing redundant variables, adopting leakage-resistant depth-block validation, and adding an independent external-well engineering reference transfer test with sparse local recalibration. The principal conclusions are:

(1) The hybrid formulation Pp = σvλp (Equation (4)) enforces dimensional consistency by construction and assigns σv a clear structural role as the pressure scale.
(2) The selected equation uses reference-scaled depth and acoustic slowness, contains no 0.505 lower threshold, and does not treat 1.05 as a discovered upper bound.
(3) The fixed selected structure achieved RMSE = 3.54 MPa and MARE = 10.02% across five contiguous depth blocks. The conservative nested estimate of the complete symbolic-selection procedure was RMSE = 4.67 MPa and MARE = 11.60%.
(4) Nested XGBoost achieved 10.51% MARE, confirming that black-box accuracy remains competitive. The contribution of symbolic regression is the explicit auditable equation and straightforward local recalibration, not universal superiority in accuracy.
(5) On the external well, the locked equation achieved MARE = 11.51% under substantial depth extrapolation but R^2^ = 0.0265, indicating limited transfer of local pressure variation. Recalibrating only the four numerical coefficients with 10 depth-spaced PP_DX engineering reference points and excluding ±20 m neighborhoods improved performance to R^2^ = 0.8684 and MARE = 4.39% on 564 remaining evaluation samples.

These results support use of the symbolic structure as an auditable, locally recalibrated screening and pressure profile model across compatible data sources, not as an unchanged universal predictor. Validation against independent direct pressure test measurements, preservation of acquisition metadata, and calibrated uncertainty quantification remain necessary before operational deployment.

Future work will focus on four priorities: preserving well and block identifiers, acquisition metadata, lithology labels, and direct pressure test records; conducting leave-one-well-out validation across multiple wells and external validation against direct formation pressure measurements; comparing wireline-only and LWD-only workflows where acquisition provenance is known; and constructing calibrated uncertainty intervals. These extensions are required before unchanged operational deployment.

## Figures and Tables

**Table 1:** Descriptive statistics and revised modeling roles of the audited inputs.

Variable	Unit	Mean	SD	Range	Modeling Role
Depth (D)	m	2249.19	572.63	1188–3215	Selected trend candidate
GR	API	93.47	12.20	54.04–136.91	Diagnostic and black-box input
DTc	μs/ft	90.95	18.83	49.64–263.04	Selected symbolic input
Vp	km/s	3.4865	0.6786	1.1587–6.1404	Exact transform of DTc; excluded from compact SR
Vs	km/s	1.7935	0.4078	0.3948–3.3883	Derived from Vp and PR; excluded
ρ	g/cm^3^	2.4356	0.1213	1.8520–2.8193	Alternative and black-box input
PR	–	0.3226	0.0168	0.2811–0.4343	Dimensionless diagnostic input
E	MPa	22,126.90	10,570.32	1023.74–80,558.67	Derived from ρ, Vs, and PR; excluded
Vsh	–	0.2360	0.1854	−0.0562–1.1885	Primarily derived from GR; excluded
UCS (shale)	MPa	76.82	26.47	8.82–197.35	Highly collinear strength indicator
σv	MPa	48.84	13.92	23.55–72.69	Physical pressure scale in Pp = σvλp
φ	°	34.69	3.71	4.22–46.05	Optional dimensionless candidate through sinφ
C	MPa	16.57	8.56	0.30–64.30	Highly correlated with UCS; excluded

**Table 2:** Symbolic regression accuracy and robustness under the revised validation design.

Estimate	Validation Design	Mean R^2^	RMSE (MPa)	MAE (MPa)	MARE (%)
Selected SR structure	Five contiguous depth blocks; fixed structure	0.3008	3.5439	2.6964	10.02
Nested SR pipeline	Structure selection inside each outer training fold	−0.1492	4.6678	3.3997	11.60
Repeated random split	100 shuffled 80:20 splits; secondary diagnostic only	0.9249 ± 0.0089	3.5235	2.5427	9.45 ± 0.37

Note: For repeated random splits, the ± values denote the standard deviation across 100 splits. RMSE and MAE are reported as arithmetic means because their split-wise standard deviations were not retained in the original aggregate output.

**Table 3:** Fold-wise performance of the selected symbolic structure.

Fold	Held-Out Depth Interval (m)	Training Size	Test Size	R^2^	RMSE (MPa)	MARE (%)
1	1188–1671	1482	376	0.4325	2.1202	11.33
2	1672–2064	1464	376	0.3755	2.3639	9.61
3	2065–2464	1462	375	0.0638	3.4389	11.67
4	2465–2839	1462	375	0.1584	4.7799	8.58
5	2840–3215	1482	375	0.4739	5.0164	8.92
Mean	–	–	–	0.3008	3.5439	10.02

**Table 4:** Target and constraint ablation comparison under consistent depth-block validation.

Model/Target	Role	Mean R^2^	RMSE (MPa)	MARE (%)
Current clipped-density expression	Former thresholded SR baseline	−0.8264	5.2921	16.83
Selected pressure ratio SR	Revised fixed structure	0.3008	3.5439	10.02
Nested pressure ratio SR	Conservative full-pipeline estimate	−0.1492	4.6678	11.60
EMW target	Best controlled target reference	0.2143	3.9681	10.57
Direct MPa target	Best controlled target reference	−0.2924	4.7628	13.43

**Table 5:** Multi-model and engineering-baseline comparison under leakage-resistant depth-block validation.

Model	Validation Basis	Mean R^2^	RMSE (MPa)	MARE (%)	Worst MARE (%)
Selected symbolic regression	Fixed selected equation	0.3008	3.5439	10.02	11.67
Nested symbolic regression	Full structure-selection pipeline	−0.1492	4.6678	11.60	12.72
XGBoost	Fully nested tuning	0.2474	3.8628	10.51	12.76
Gradient Boosting	Fully nested tuning	0.1421	4.1696	11.04	14.49
Random Forest	Fully nested tuning	0.0447	4.3290	12.06	14.06
SVR	Fully nested tuning	−0.6644	5.8608	15.85	24.61
Current clipped-density SR	Former expression	−0.8264	5.2921	16.83	23.17
Hydrostatic	Engineering reference	−2.0315	7.8267	19.23	33.66
Eaton-type	Engineering reference	−4.6029	10.3915	24.92	46.33

**Table 6:** External-well zero-shot transfer and sparse local coefficient recalibration.

Strategy	Calibration Points	Evaluation N	R^2^	RMSE (MPa)	MAE (MPa)	MARE (%)
Zero-shot locked equation	0	974	0.0265	7.0024	5.5723	11.51
Sparse local recalibration	5	769	0.7772	3.5193	2.7176	5.65
Sparse local recalibration	10	564	0.8684	2.8431	2.1108	4.39
Sparse local recalibration	20	154	0.9155	2.8056	1.9709	3.83

Note: Zero-shot results use the original locked coefficients. For recalibration rows, all samples within ±20 m of every selected PP_DX reference point were excluded from evaluation; only the four numerical coefficients of the fixed structure were re-estimated.

## Data Availability

The data used in this study are available from the corresponding author upon reasonable request, subject to project and data-use restrictions. The primary calibration dataset does not preserve well identifiers, block identifiers, acquisition-tool metadata, lithology labels, or direct pressure-test identifiers. The separate external-well table was used only for external engineering-reference validation; its PP_DX target is D-exponent derived and is not an MDT/RFT/DST direct-pressure measurement.

## Appendix A

The benchmark models were tuned independently within each outer training set. Table A1 reports the candidate search spaces and fixed implementation settings used to ensure a transparent and consistent comparison.

**Table A1:** Hyperparameter search spaces and tuning settings for the benchmark models.

Model	Candidate Hyperparameter Values	Fixed Settings
Random Forest	n_estimators: 200, 400; max_depth: 8, None; min_samples_leaf: 1, 3; max_features: sqrt, 1.0	random_state = 42; bootstrap = True
Gradient Boosting	n_estimators: 100, 200; learning_rate: 0.03, 0.05; max_depth: 2, 3; min_samples_leaf: 1, 3	random_state = 42; loss = squared_error
XGBoost	n_estimators: 100, 200; learning_rate: 0.03, 0.05; max_depth: 2, 3; subsample: 0.8, 1.0	colsample_bytree = 0.9; reg_lambda = 1.0; objective = reg:squarederror; random_state = 42
SVR	C: 1, 10, 100; gamma: scale, 0.01; epsilon: 0.05, 0.10	RBF kernel; StandardScaler fitted only on the corresponding training partition

For all four benchmark models, hyperparameter selection used inner contiguous depth-block validation within each outer training set; the corresponding outer held-out block was not used for model selection.
